# Implementation of a Delirium Bundle for Pediatric Intensive Care Patients

**DOI:** 10.3389/fped.2022.826259

**Published:** 2022-02-07

**Authors:** Jörg Michel, Elena Schepan, Michael Hofbeck, Juliane Engel, Alexander Simma, Felix Neunhoeffer

**Affiliations:** Department of Pediatric Cardiology, Pulmonology and Pediatric Intensive Care Medicine, University Children's Hospital Tübingen, Tübingen, Germany

**Keywords:** PICU, sedation, analgesia, withdrawal, critical care, delirium, post-intensive care syndrome, PICU delirium bundle

## Abstract

**Background and Objective:**

Delirium represents the most common form of acute cerebral dysfunction in critical illness. The prevention, recognition, and treatment of delirium must become the focus of modern pediatric intensive care, as delirium can lead to increased morbidity and mortality. The aim of this study is to evaluate the impact of a delirium bundle consisting of mainly non-pharmacological measures.

**Material and Methods:**

This is a pre-/post-implementation study in an interdisciplinary pediatric intensive care unit of a tertiary care university hospital. In the pre-implementation period, pediatric intensive care delirium was monitored using the Sophia Observation withdrawal Symptoms and Pediatric Delirium scale. After introduction of a delirium bundle consisting of non-pharmacological prevention and treatment measures a period of 4 months was interposed to train the PICU staff and ensure that the delirium bundle was implemented consistently before evaluating the effects in the post-implementation period. Data collection included prevalence of delirium and withdrawal, length of PICU stay, duration of mechanical ventilation, and cumulative dose of sedatives and analgesics.

**Results:**

A total of 792 critically ill children aged 0–18 years were included in this study. An overall delirium prevalence of 30% was recorded in the pre-implementation group and 26% in the post-implementation group (*p* = 0.13). A significant reduction in the prevalence of pediatric delirium from was achieved in the subgroup of patients under 5 years of age (27.9 vs. 35.8%, *p* = 0.04) and in patients after surgery for congenital heart disease (28.2 vs. 39.5%, *p* = 0.04). Young age, length of PICU stay, and iatrogenic withdrawal syndrome were found to be risk factors for developing delirium.

**Conclusions:**

Based on a validated delirium monitoring, our study gives new information regarding the prevalence of pediatric delirium and the characteristics of intensive care patients at risk for this significant complication. Especially young patients and patients after surgery for congenital heart disease seem to benefit from the implementation of non-pharmacological delirium bundles. Based on our findings, it is important to promote change in pediatric intensive care—toward a comprehensive approach to prevent delirium in critically ill children as best as possible.

## Introduction

Delirium in pediatric intensive care unit patients (PICU delirium) is a complication of critical illness affecting attention, cognition, and awareness and is associated with a poor outcome. PICU delirium can develop within a short period of time. The hypoactive delirium is distinguished from the hyperactive and the mixed form, and symptoms can fluctuate throughout the day (1). Delirium is a result of pre-existing risk factors, underlying disease and medical conditions, iatrogenic drug exposure, and environmental factors during the intensive care stay (2). Independent risk factors are young age, developmental delay, benzodiazepine exposure, and mechanical ventilation (3). The prevalence of PICU delirium is reported to range from 17 to 66% (2, 4). In children, hypoactive delirium and the mixed form are most common, and last for several days (5–8). There are significant associations between PICU delirium, increased duration of mechanical ventilation, length of stay, used resources, and medical costs (2, 5, 8, 9). Delirium is also independently associated with mortality in children (2). Data to long-term outcomes associated with pediatric delirium are rare. Two authors found an association between delirium during the PICU stay with decline in health-related quality of life (10, 11). Evidence on measures to prevent and manage delirium is urgently needed. There are few reports of low quality on pharmacological management of pediatric delirium with typical and atypical antipsychotic drugs which led to improvement in delirium symptoms, but side effects such as extrapyramidal symptoms, heavy sedation, and prolonged corrected QT (QTc) interval were common (12–14). It remains unclear if antipsychotic use reduces overall delirium prevalence or effectively treats hypoactive or mixed delirium (15). The risks associated with antipsychotic management may not outweigh the risks in all patients, however, in hyperactive delirium the benefits may outweigh the risks. As alternative to pharmacological management, the bundle approach, multicomponent delirium interventions, seems to be promising. Based on evidence of delirium bundle in the adult population, bundle intervention may decrease the incidence of delirium as well in the pediatric population (16, 17). Nevertheless, a recent published meta-analysis failed to support that bundle interventions are effective in reducing ICU delirium prevalence and duration, although, it supported that bundle interventions are effective in reducing the proportion of patient-days with coma, hospital length of stay, and 28-day mortality (18).

When creating developmentally appropriate bundle for the pediatric population, caregivers should focus on modifiable risk factors. Modifiable risk factors are clinical variables such as mechanical ventilation, choice of sedating medications, especially reduction of benzodiazepine exposure, reduction of anticholinergic drugs, administration of red blood cells, physical restraints, and good nutrition (2, 5, 6, 8, 9, 11, 17). A structured approach to introduce delirium bundle at the PICU may prevent delirium. We have sustainably implemented a functioning nurse-driven analgesia and sedation protocol on our PICU, that was feasible and safe and reduces length of PICU stay, cumulative dose of benzodiazepines and withdrawal symptoms (19–23).

The aim of this study was to evaluate the impact of a delirium bundle consisting of mainly non-pharmacological measures in a pediatric intensive care unit of a tertiary center.

## Materials and Methods

### Study Design

**Figure 1:**
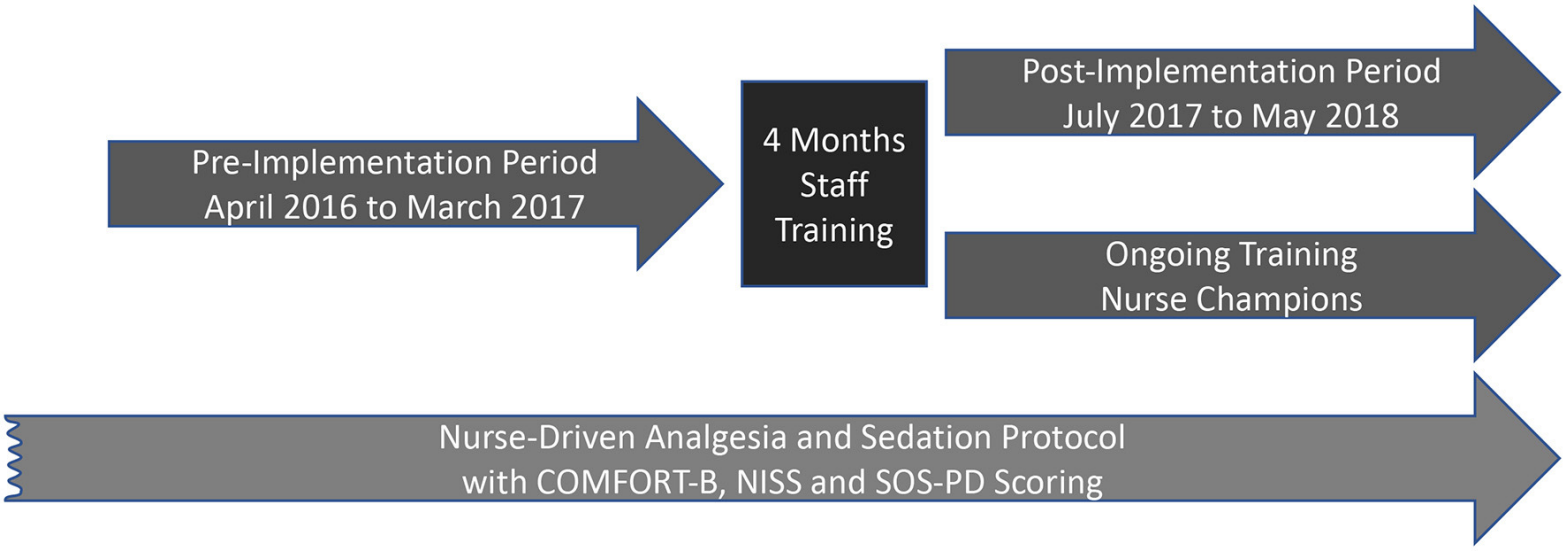
Conceptual design of the study.

**Figure 2:**
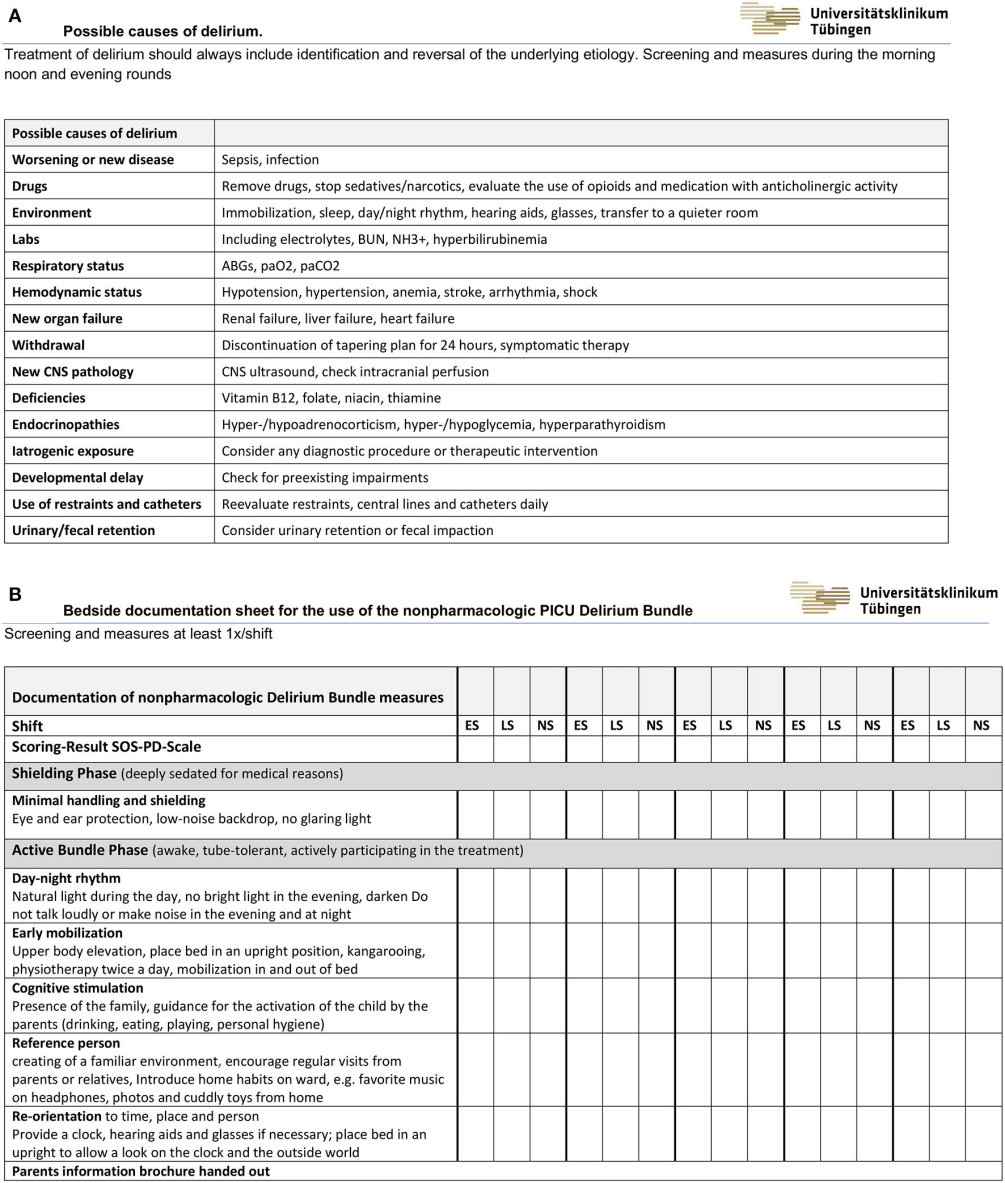
**(A)** Possible causes of pediatric delirium. **(B)** Bedside checklist and documentation sheet for the non-pharmacologic measures of the PICU Delirium Bundle (ES, Early Shift; LS, Late Shift; NS, Night Shift).

This is a non-randomized, monocentric, pre-/post-implementation study. In the pre-implementation period (January 2016–February 2017), PICU delirium was monitored using the Sophia Observation withdrawal Symptoms and Pediatric Delirium (SOS-PD) scale (24, 25). In March 2017 a delirium bundle consisting of non-pharmacological prevention and treatment measures was introduced. A period of 4 months was interposed to train the PICU staff and ensure that the delirium bundle was implemented consistently by verifying that delirium scoring as well as bedside documentation of non-pharmacologic measures were regularly used and filled out before evaluating the effects in the post-implementation period from July 2017 to May 2018 (Figures 1, 2). Clinical data of our patients including age, gender, weight, diagnosis, length of PICU stay, duration of mechanical ventilation, levels of sedation and analgesia, incidence and duration of delirium and withdrawal, cumulative dose of sedatives and analgesics, and safety-relevant events due to the application of the bundle were collected from the patient data management system (IntelliSpace Critical Care and Anesthesia, Koninklijke Philips N.V., the Netherlands). All parameters were routinely assessed and automatically calculated by the patient data management system in intervals of 8 h. At the end of the study, the data were extracted from the patient data management system. The study protocol was approved by the local ethics committee (650/2015BO1).

### Study Location and Population

The study was conducted at a 14-bed interdisciplinary PICU at a University Children's Hospital. The ratio of registered nursing staff to patients was between 1:1 and 1:2, the ratio of registered medical doctors to patients was between 1:5 and 1:7. The hospital is a tertiary referral center including active departments of pediatric cardiovascular surgery, pediatric surgery, pediatric neurosurgery, transplantation, trauma, as well as hematology and oncology services. All patients between 0 and 18 years of age admitted to the PICU with a length of stay of at least 24 h were enrolled in this study. Exclusion criteria were diagnosed encephalitis, or death. In addition, periods of very deep sedation defined by a COMFORT Behavior Scale (COMFORT B) (26) < 11, coma, or continuous neuromuscular blockade were not considered.

### Nurse Driven Analgesia, Sedation and Withdrawal Protocol, and Drugs and Routes

During the entire study period, sedation and analgesia medication was titrated to attain a COMFORT-B level of 12–18 and a nurse interpretation sedation scale (NISS) level of 2 (adequate sedation) following the updated version of our institutional standardized, goal-directed nurse-driven analgesia, and sedation protocol, which has been described in detail previously (20). The standard therapy during the study period consisted of continuous i.v. infusion of opioids (morphine [5–100 μg·kg^−1^·h^−1^; starting dose 30 μg·kg^−1^·h^−1^] ≤ 2 years of age and fentanyl [0.1–6.0 μg·kg^−1^·h^−1^; starting dose 0.5 μg·kg^−1^·h^−1^] > 2 years) and continuous i.v. infusion of clonidine (0.5–2 μg·kg^−1^·h^−1^). The updated version of the analgesia and sedation protocol did not involve the routinely administration of midazolam. Oral/rectal chloral hydrate (up to 6 × 25 mg·kg^−1^·day^−1^) and oral melatonin (3–7 mg·day^−1^) were administered additionally according to our PICU guideline. However, to protect patients' safety in case of undersedation the attending intensivist could deviate from the updated sedation protocol at any time. During weaning from analgesia and sedation medication, children were monitored regarding withdrawal symptoms and delirium using the SOS-PD scale (24, 25, 27). The medication tapering plan provided reduction of opioids and benzodiazepines by 50% of the dose every 24 h in case of therapy lasting 5 days or less, and by 10–20% every 24 h in case of therapy longer than 5 days. A SOS score of ≥4 indicates withdrawal, and the medication tapering plan was paused for 24 h.

### Delirium Scoring and Management Pre-implementation Period

We had decided in advance to use the SOS-PD scale in this study and in daily clinical practice because this scale, in contrast to Cornell Assessment of Pediatric Delirium (CAP-D), measures both delirium and withdrawal and discriminates between them. Delirium screening was performed and documented in the patient data management system (PDMS) at least every 8 h. The SOS-PD scale, the SOS scale, extended with a pediatric delirium (PD) component, has promising validity, and reliability (24, 25). The intraclass correlation coefficient (ICC) of paired nurse-researcher observations was 0.90 (95% CI: 0.70–0.96) (28). The sensitivity was 96.8% (95% CI: 80.4–99.5%) and the specificity was 92.0% (95% CI: 59.7–98.9%) (25). Pearson coefficient between the SOS-PD scale and the CAP-D was 0.89 (CI 95%, 0.82–0.93; *p* < 0.001). A very good agreement (Kappa = 1; *p* < 0.001) between the two scales was identified (29). Compared to the psychiatrist diagnosis, the overall sensitivity was 92.3% with a specificity of 96.5% (25). No prophylaxis measures were routinely performed, delirium management was carried out according to the decision of the responsible physician.

### Delirium Scoring and Management Post-implementation Period

An interprofessional team consisting of nurses, intensivists, psychiatrists, and pharmacists developed the PICU delirium bundle and a training plan to improve PICU staff education. The team first conducted a review of literature regarding evidence-based assessment and management of PICU delirium to develop the non-pharmacologic delirium bundle. Little literature was available on detection, prevention, and management of delirium in children in the intensive care unit at the time the bundle was designed. Most studies recommended family support and family presence in the ICU, operational, and environmental modifications and improving communication with families. We have selected the following as the most important measures for our setting: Providing a calm and reassuring environment (30–34), providing pictures of the family of home and personal cuddly toys, having favorite toys, music and personal items ready, like a mother's t-shirt (33, 35–37), avoiding physical restraints (37, 38), children who need glasses or hearing aids should wear them when possible (34, 39), creating an schedule of daytime activities and nighttime sleep, placing bed in a upright position when child tolerates, discourage sleep during the daytime except for scheduled naps or quiet rest times (40), having a calendar and clock for date and time identification (37), using a dim light at night (41), using eye masks to block light during sleep and earplugs to block noise (40, 42), allowing the view outside to determine the time of day (36), and guidance for parents to reorient their child to person, place, time, and reason for being in the hospital (43–45). In addition to the existing pain, sedation, withdrawal and delirium assessment instruments, the designed bundle comprises non-pharmacologic prevention strategies, identification of potential etiologies, and treatment measures. To identify and reverse the underlying etiology of pediatric delirium we developed a checklist, based on the mnemonic “I WATCH DEATH,” to screen for possible causes during the morning, noon, and evening rounds (Figure 2A) (46). Pharmacologic treatment is not part of the bundle. A period of 4 months was interposed to train the PICU staff and ensure that the delirium bundle was implemented consistently. Nursing and physician staff participated in several 1-h educational sessions about delirium causes, consequences, prevention, identification, and management. PICU staff received training on how to conduct and document delirium scoring. During the sessions, sample videos of patients with delirium symptoms and patients with withdrawal symptoms were demonstrated, which were used to practice scoring and explain the differentiation between withdrawal symptoms and delirium. Furthermore, the documentation forms of the non-pharmacological measures, the differences between the shielding phase and the active bundle phase, and the measures in these two phases were explained. The SOS-PD scale had been introduced and trained before the study. SOS-PD scale and PICU delirium bundle were available on the PDMS, and on bedside charts. Nurse champion were available to answer questions, provide assistance and solve problems. Resident physicians participated in 1-h educational sessions at the beginning of their PICU rotation. The bundle is divided into two phases. The “shielding phase” is used for children who need to be deeply sedated for medical reasons and involves the complete shielding of noise and light through eye and ear protection. The second phase is applied to all other children who may be awake and tube-tolerant. This phase includes the creation of a day-night rhythm, mobilization in bed and, if possible, out of bed, cognitive stimulation by parents after guidance, choosing of reference persons in the team, and involvement of the parents in the care of their children. Required hearing aids and glasses were provided at an early stage. Parents were encouraged to bring along music, photos, and cuddly toys from home. To improve the children's ability to reorient themselves, care was taken to ensure that the head of the bed was placed in an upright position and that they had a view of a clock and the outside world. At the beginning of the intensive stay, the parents were given a brochure explaining withdrawal and delirium and providing advice on how to deal with their children (Supplementary Material). A printed version of the brochure was available at each patient's bedside. The bedside documentation sheet of the non-pharmacological PICU delirium bundle measures is shown in Figure 2. If delirium with severe agitation or hyperactive symptoms persisted despite interventions to address potential causes, pharmacologic antipsychotic treatments was started in individual cases with low-dose levomepromazine, an aliphatic phenothiazine neuroleptic drug (0.1 mg·kg^−1^) (47).

### Statistical Analysis

Statistical analysis and the creation of charts were performed using SigmaPlot (Version 13 for Windows, Systat Software, Inc., San Jose, CA, US) and SPSS (Version 24, IBM, Armonk, NY, US). Normality was assessed using the Shapiro-Wilk test. Data are presented as median [interquartile range (IQR)]. For statistical analysis Student's *t*-test and the Mann–Whitney Rank Sum test was applied, depending on whether the data were normally distributed. Categorical variables were compared using Two-tailed Fisher's exact test. A probability of *p* < 0.05 was defined as statistically significant. To compare the amount of opioids given in patients ≤ 2 years of age and patients >2 years of age, we converted opioids to morphine equivalents; the equipotency ratio of i.v. fentanyl to i.v. morphine was calculated as 1:80.

## Results

**Figure 3:**
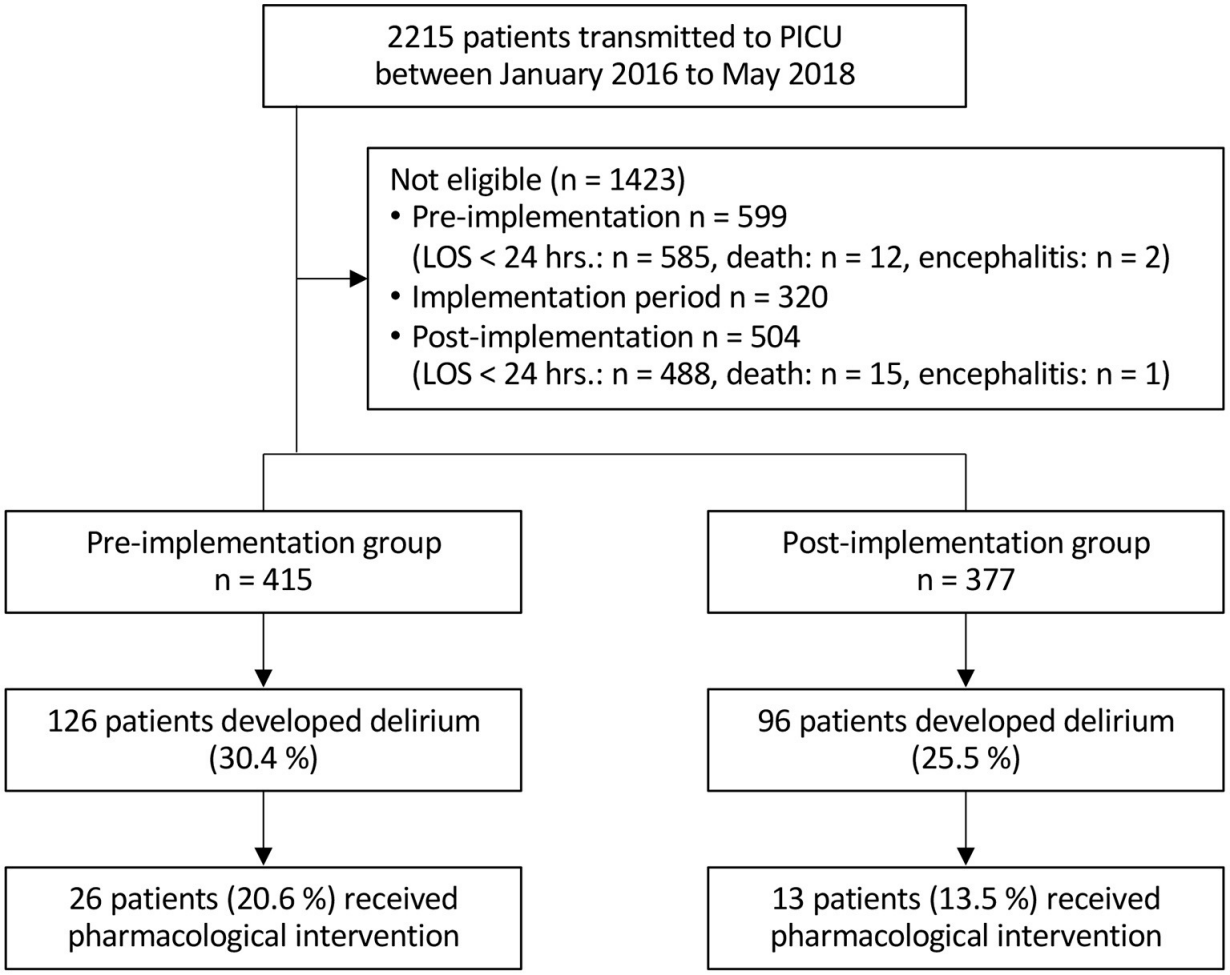
CONSORT flow diagram (LOS, Length of stay).

A total of 792 critically ill children aged 0–18 years were included in this study (415 in the pre-implantation group, 377 in the post-implantation group) (Figure 3). Table 1 summarizes the patients' characteristics. There were no significant differences between the two groups in gender (m/f 224/191 vs. 205/172, *p* = 0.94), age (11.6 [2.6–55.8] vs. 15.1 [2.8–64.7] months, *p* = 0.15), weight (8.4 [4.0–17.0] vs. 9.0 [4.0–17.0] kg, *p* = 0.51), duration of ventilation (2.8 [0.7–11.6] vs. 2.3 [0.6–9.6] days, *p* = 0.33), and length of PICU stay (4.0 [1.9–12.8] vs. 3.9 [1.9–11.0] days, *p* = 0.25). In the post-implementation group, significantly fewer patients received midazolam (72.2 vs. 39.3%, *p* < 0.01) and opioids (85.8 vs. 78%, *p* = 0.01). An overall delirium prevalence of 30.4% with a median duration of 0.44 [0.0–6.6] days was recorded in the pre-implementation group and 25.5% with a median duration of 0.46 [0.0–3.1] days in the post-implementation group (prevalence *p* = 0.13; duration *p* = 0.29) (Table 2).

**Table 1:** Differences of patients' characteristics between the pre-implementation group and post-implementation group.

**Parameter**		**Pre**	**Post**	***p*-value**
Sex (m/f)	*n* (%)	224/191 (54.0/46.0%)	205/172 (54.4/45.6%)	0.94
Age (mo)	Median [IQR]	11.6 [2.6–55.8]	15.1 [2.8–64.7]	0.15
Weight (kg)	Median [IQR]	8.4 [4.0–17.0]	9.0 [4.0–17.0]	0.51
Ventilator days	Median [IQR]	2.8 [0.7–11.6]	2.3 [0.6–9.6]	0.33
Length of PICU stay (d)	Median [IQR]	4.0 [1.9–12.8]	3.9 [1.9–11.0]	0.25
Cumulative opioids (μg/kg)	Median [IQR]	968 [269–3,939]	580 [78–3,685]	0.09
Patients w/o opioids	*n* (%)	59 (14.2%)	83 (22.0%)	0.01
Cumulative midazolam (mg/kg)	Median [IQR]	2.2 [0.0–14.2]	0.0 [0.0–3.5]	0.55
Patients w/o midazolam	*n* (%)	157 (37.8%)	229 (60.7%)	<0.01
Cumulative clonidine (μg/kg)	Median [IQR]	0.0 [0.0–152.9]	0.0 [0.0–126.6]	<0.01
Patients w/o clonidine	*n* (%)	213 (51.3%)	191 (50.7%)	0.89

*IQR, interquartile range; PICU, pediatric intensive care unit*.

**Table 2:** Prevalence of delirium and withdrawal symptoms in all patients, in patients grouped by age, and in patients grouped by disease.

**Parameter**	**Pre**	**Post**	***p*-value**
**All patients**			
Patients with withdrawal symptoms	63/415 patients (15.2%)	40/377 patients (10.6%)	0.06
Patients with delirium	126/415 patients (30.4%)	96/377 patients (25.5%)	0.13
Duration of delirium (d)	0.44 [0.0–6.6]	0.46 [0.0–3.1]	0.29
Patients with delirium needing pharmacological intervention	26 patients (20.6%)	13 patients (13.5%)	0.07
**Age** **<** **60 months**			
Patients with withdrawal symptoms	60/318 patients (18.9%)	32/276 patients (11.6%)	0.02
Patients with delirium	114/318 patients (35.8%)	77/276 patients (27.9%)	0.04
Duration of delirium (d)	0.8 [0.0–7.1]	0.4 [0.0–3.0]	0.21
**Age** **>** **60 months**			
Patients with withdrawal symptoms	3/97 patients (3.1%)	8/101 patients (7.9%)	0.21
Patients with delirium	15/97 patients (15.5%)	18/101 patients (17.8%)	0.71
Duration of delirium (d)	0.1 [0.0–1.9]	1.1 [0.0–3.7]	0.66
**Patients with CHD**			
Patients withdrawal symptoms	34/185 patients (18.4%)	18/131 patients (13.7%)	0.29
Patients with delirium	73/185 patients (39.5%)	37/131 patients (28.2%)	0.04
Duration of delirium (d)	0.3 [0.0–7.2]	0.7 [0.0–4.3]	0.91
**Patients after surgery (other than CHD)**			
Patients withdrawal symptoms	22/130 patients (16.9%)	15/168 patients (8.9%)	0.05
Patients with delirium	35/130 patients (26.9%)	41/168 patients (24.4%)	0.69
Duration of delirium (d)	2.3 [0.0–10.0]	0.0 [0.0–1.8]	0.01
**Patients with other diseases (e.g., hematological, oncological**, **neuropediatric, and nephrological)**			
Patients withdrawal symptoms	7/100 patients (7.0%)	7/78 patients (9.0%)	0.78
Patients with delirium	18/100 patients (18.0%)	18/78 patients (23.1%)	0.45
Duration of delirium (d)	0.12 [0.0–2.6]	1.7 [0.0–5.2]	0.30

*CHD, congenital heart disease*.

In the subgroup analysis of patients younger than 5 years, a significant reduction in the prevalence of delirium was recorded after the introduction of delirium bundles (35.8 vs. 27.9%, *p* = 0.04) (Table 2). The median duration of delirium also showed a decreasing trend, but was not statistically significant (0.8 [0.0–7.1] vs. 0.4 [0.0–3.0], *p* = 0.21). In this subgroup, there were no significant differences between the pre-implementation and post-implementation group in (m/f 175/143 vs. 146/130, *p* = 0.62), age (5.8 [0.8–23.7] vs. 5.9 [0.8–20.5] months, *p* = 0.61), weight (5.7 [3.5–11.0] vs. 6.0 [3.5–11.1] kg, *p* = 0.94), duration of ventilation (4.3 [0.9–13.9] vs. 3.1 [0.7–11.6] days, *p* = 0.16), and length of PICU stay (5.4 [2.5–15.1] vs. 4.0 [1.9–11.8] days, *p* = 0.10) (Table 3).

**Table 3:** Differences of patients' characteristics between the pre-implementation group and post-implementation group in patients aged 60 months and younger.

**Parameter**		**Pre**	**Post**	***p*-value**
Sex (m/f)	*n* (%)	175/143 (55.0/45.0%)	146/130 (52.9/47.1%)	0.62
Age (mo)	Median [IQR]	5.8 [0.8–23.7]	5.9 [0.8–20.5]	0.61
Weight (kg)	Median [IQR]	5.7 [3.5–11.0]	6.0 [3.5–11.1]	0.94
Ventilator days	Median [IQR]	4.3 [0.9–13.9]	3.1 [0.7–11.6]	0.16
Length of PICU stay (d)	Median [IQR]	5.4 [2.5–15.1]	4.0 [1.9–11.8]	0.10
Cumulative opioids (μg/kg)	Median [IQR]	1,372 [474–5,643]	710 [139–4,096]	0.04
Patients w/o opioids	*n* (%)	40 (12.6%)	53 (19.2%)	0.03
Cumulative midazolam (mg/kg)	Median [IQR]	3.1 [0.0–19.1]	0.0 [0.0–5.1]	0.52
Patients w/o midazolam	*n* (%)	100 (31.4%)	158 (57.2%)	<0.01
Cumulative clonidine (μg/kg)	Median [IQR]	19.3 [0.0–249.6]	13.9 [0.0–137.9]	<0.01
Patients w/o clonidine	*n* (%)	141 (44.3%)	129 (46.7%)	0.56
Performed scorings per day	Median [IQR]	3.0 [2.0–3.9]	2.3 [1.8–2.8]	<0.001

*IQR, interquartile range; PICU, pediatric intensive care unit*.

The prevalence of delirium was also significantly reduced in the subgroup of patients after surgery for congenital heart disease from 39.5 to 28.2% (*p* = 0.04) (Table 2). Again, there were no significant differences between both groups in (m/f 104/81 vs. 73/59, *p* = 0.91), age (4.7 [0.6–25.0] vs. 5.7 [0.8–30.8] months, *p* = 0.22), weight (5.4 [3.5–10.9] vs. 6.0 [3.5–10.2] kg, *p* = 0.74), duration of ventilation (2.8 [0.8–13.9] vs. 2.2 [0.7–12.0] days, *p* = 0.81), and length of PICU stay (5.0 [2.4–15.9] vs. 4.8 [1.9–13.0] days, *p* = 0.64) (Table 4).

**Table 4:** Differences of patients' characteristics between the pre-implementation group and post-implementation group in patients with congenital heart disease.

**Parameter**		**Pre**	**Post**	***p*-value**
Sex (m/f)	*n* (%)	104/81 (56.2/43.8%)	73/59 (55.3/44.7%)	0.91
Age (mo)	Median [IQR]	4.7 [0.6–25.0]	5.7 [0.8–30.8]	0.22
Weight (kg)	Median [IQR]	5.4 [3.5–10.9]	6.0 [3.5–10.2]	0.74
Ventilator days	Median [IQR]	2.8 [0.8–13.9]	2.2 [0.7–12.0]	0.81
Length of PICU stay (d)	Median [IQR]	5.0 [2.4–15.9]	4.8 [1.9–13.0]	0.64
Cumulative opioids (μg/kg)	Median [IQR]	1,347 [481–4,971]	899 [212–4,652]	0.59
Patients w/o opioids	*n* (%)	9 (4.9%)	21 (16.0%)	<0.01
Cumulative midazolam (mg/kg)	Median [IQR]	2.9 [0.0–16.8]	0.0 [0.0–6.3]	0.36
Patients w/o midazolam	*n* (%)	60 (32.4%)	72 (55.0%)	<0.01
Cumulative clonidine (μg/kg)	Median [IQR]	26.0 [0.0–187.5]	24.0 [0.0–132.7]	0.12
Patients w/o clonidine	*n* (%)	78 (42.2%)	53 (40.5%)	0.82

*IQR, interquartile range; PICU, pediatric intensive care unit*.

**Figure 4:**
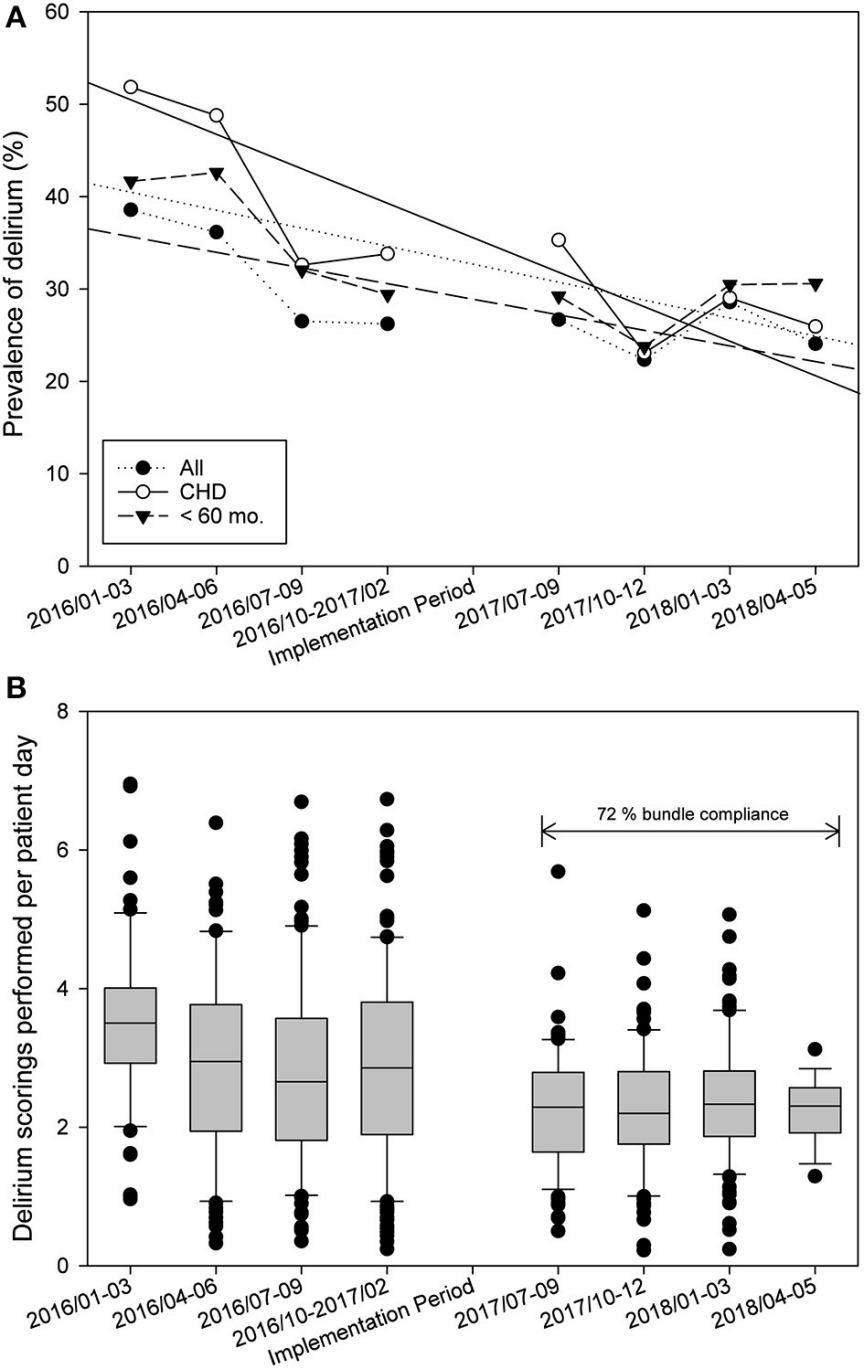
Statistical process control chart of time [**(A)** prevalence of delirium in all patients, in patients after surgery for congenital heart disease and in patients aged 60 months and younger. **(B)** Delirium scorings performed per day].

Using logistic regression analysis, young age (OR = 0.995; 95% CI: 0.992–0.999; *p* = 0.02), length of PICU stay (OR = 1.035; 95% CI: 1.010–1.061; *p* < 0.01), and iatrogenic withdrawal syndrome (OR = 54.052; 95% CI: 19.096–152.999; *p* < 0.01) were found to be risk factors for developing delirium (Table 5). Patients with delirium were significantly younger (7.3 [1.9–33.4] vs. 22.0 [3.0–78.5] months, *p* < 0.01), had lower weight (6.5 [3.8–13.0] vs. 10.7 [4.1–19.0] kg, *p* < 0.01), had longer duration of ventilation (10.2 [3.4–22.9] vs. 1.2 [0.3–5.0] days, *p* < 0.01) and longer length of PICU stay (12.9 [6.0–26.3] vs. 2.9 [1.7–5.9] days, *p* < 0.01). They received more opioids (cumulative dose 4,851 [1,600–12,073] vs. 491 [56–1,409] μg·kg^−1^, *p* < 0.01) midazolam (cumulative dose 13.3 [1.9–56.6] vs. 0.0 [0.0–2.5] mg·kg^−1^, *p* < 0.01), and clonidine (cumulative dose 211.9 [55.6–728.2] vs. 0.0 [0.0–28.1] μg·kg^−1^, *p* < 0.01) (Table 6). In the pre-implementation group, 26 patients (20.6%) who developed delirium and showed severe agitation or hyperactive delirium symptoms despite non-pharmacological measures received pharmacologic therapy, compared with 13 patients (13.5%) in the post-implementation group (*p* = 0.07). Patients who received pharmacologic delirium therapy had longer duration of ventilation (21.1 [10.4–50.7] vs. 7.0 [1.9–17.3] days, *p* < 0.001), longer length of PICU stay (25.9 [14.3–66.1] vs. 11.0 [4.7–22.7] days, *p* < 0.001), longer duration of delirium (9.6 [3.7–44.5] vs. 0.1 [0.0–2.0] days, *p* < 0.001), and a higher prevalence of iatrogenic withdrawal syndrome (33/39 [84.6%] vs. 66/183 [36.1%], *p* < 0.0001). No difference was observed in age (12.3 [3.8–37.0] vs. 6.2 [1.5–32.2] months, *p* = 0.14) and weight (7.2 [5.0–12.0] vs. 6.1 [3.7–13.0] kg, *p* = 0.29). Scoring was performed a median of 3.0 [2.0–3.9] times per patient day during the pre-implementation phase and 2.3 [1.8–2.8] in the post-implementation period (*p* < 0.001, Figure 4). During the post-implementation period, the adherence to the bundle was randomly checked and showed an average compliance rate of 72% for the bundle. No adverse events associated with the PICU delirium bundles were reported.

**Table 5:** Odds ratios for effects of sex, age, length of PICU stay, duration of mechanical ventilation, and withdrawal symptoms on development of delirium.

**Variable**	**Regression coefficient**	**Standard error**	**Odds ratio**	**95%-CI**	***p*-value**
Sex (male/female)	0.110	0.211	1.117	0.738–1.690	0.60
Age (mo)	−0.005	0.002	0.995	0.992–0.999	0.02
Length of PICU stay (d)	0.035	0.013	1.035	1.010–1.061	<0.01
Mechanical ventilation (d)	0.009	0.013	1.009	0.984–1.036	0.48
Withdrawal symptoms	3.990	0.531	54.052	19.096–152.999	<0.01

*CI, Confidence interval; PICU, pediatric intensive care unit*.

**Table 6:** Differences of patients' characteristics between patients with delirium and patients without delirium.

**Parameter**		**Pre**	**Post**	***p*-value**
Sex (m/f)	*n* (%)	123/99 (55.4/44.6%)	306/264 (54.6/45.4%)	0.69
Age (mo)	Median [IQR]	7.3 [1.9–33.4]	22.0 [3.0–78.5]	<0.01
Weight (kg)	Median [IQR]	6.5 [3.8–13.0]	10.7 [4.1–19.0]	<0.01
Ventilator days	Median [IQR]	10.2 [3.4–22.9]	1.2 [0.3–5.0]	<0.01
Length of PICU stay (d)	Median [IQR]	12.9 [6.0–26.3]	2.9 [1.7–5.9]	<0.01
Cumulative opioids (μg/kg)	Median [IQR]	4,851 [1,600–12,073]	491 [56–1,409]	<0.01
Patients w/o opioids	*n* (%)	9 (7.4%)	133 (23.3%)	<0.01
Cumulative midazolam (mg/kg)	Median [IQR]	13.3 [1.9–56.6]	0.0 [0.0–2.5]	<0.01
Patients w/o midazolam	*n* (%)	46 (20.7%)	340 (59.6%)	<0.01
Cumulative clonidine (μg/kg)	Median [IQR]	211.9 [55.6–728.2]	0.0 [0.0–28.1]	<0.01
Patients w/o clonidine	*n* (%)	31 (14.0%)	373 (65.4%)	<0.01

*IQR, interquartile range; PICU, pediatric intensive care unit*.

## Discussion

Delirium in critically ill children is a serious problem that affects short- and long-term morbidity and mortality, among other outcomes (2, 5, 6, 9, 11). Many studies are now available on modifiable and non-modifiable risk factors for pediatric delirium. Although, the efficacy of multicomponent delirium interventions has been demonstrated in adult intensive care patients, there have been few studies on the efficacy of delirium bundles in pediatric intensive care patients (16, 17, 48). One challenge is certainly to adapt and implement these interventions in PICUs (48). Pediatric delirium screening is not being performed consistently in most PICUs internationally, regular monitoring of delirium with validated assessment tools is practiced in only 25–40% of PICUs (49, 50). Knowledge about delirium among PICU staff is still insufficient, and sustainably designed training programs are urgently needed (51). Interventions should focus on validated sedation, pain, withdrawal, and delirium screening tools, identification of potential delirium risk factors, analgesia, and sedation protocols, avoidance of deliriogenic medications, reorientation measures, modification of environmental factors, early mobilization, family empowerment and engagement, and sleep promotion (16, 17).

We have successfully implemented and use a nurse-controlled analgesia and sedation protocol in our clinical routine for several years with validated scoring instruments for sedation, pain, withdrawal, and delirium (20). Building on this, we created a delirium bundle in a multi-professional team, developed a sustainable training concept and named nurse champions. This is one of the first prospective studies addressing the implementation of a delirium bundle in critically ill children in a before and after study design. The overall prevalence of delirium showed a statistically non-significant reduction from 30% before implementation to 26% after implementation. Compared with other studies, the prevalence of delirium is somewhat lower in our collective, possibly influenced by our analgesia and sedation protocol, and the routine scoring of sedation, pain, withdrawal, and delirium. The reported prevalence of delirium in PICU patients is up to 57% in pediatric postoperative cardiac surgery patients (4, 6, 8, 9, 11, 52). The most vulnerable patient group appears to be young children after cardiothoracic surgery, and in this group our non-pharmacological delirium bundle was most effective (9, 53). In patients under 5 years of age and especially in patients after surgery for congenital heart disease a significant reduction in the prevalence of pediatric delirium from 36 to 28% and from 40 to 29% was found. In agreement with other studies, we found length of PICU stay, iatrogenic withdrawal, and young age to be risk factors for developing delirium (2, 4, 6, 8, 9).

Simone et al. (17) described that the prevalence of delirium can be reduced in a subgroup of young patients and patients after surgery for congenital heart disease by implementing non-pharmacological prevention and therapy measures during sequential implementation of delirium, sedation, and early mobility protocols over a 22-month period. Delirium screening compliance was 95% throughout the study, compliance rates for bundle components were not reported (17). Delirium scoring was performed a median of 3.0 [2.0–3.9] times per patient day during pre-implementation period and 2.3 [1.8–2.8] during post-implementation period. The average compliance rate for the bundle was 72%, single components of the bundle were not examined. The high compliance rate of delirium scorings and for the bundle could be the result of the extensive ongoing training program and the presence of nurse champions. In another study, Franken et al. (48) found no difference in average CAP-D scores following a non-pharmacologic nursing bundle implementation, compared to a retrospective control group. Screening compliance was low with 6–9%, with only few positive CAP-D screening results, compliance rates for bundle components were not reported (48). Implementing delirium screening and delirium bundle in a complex environment like a PICU is a great challenge, but universal delirium screening, prevention, and management are feasible and sustainable and can become standard care on a PICU, if you involve all PICU team members and have a long breath.

Important limitations of this study are the single-center and the study design, the absence of randomization in our study population, and missing blinding of the involved health care professionals. Therefore, we cannot exclude, that the delirium prevalence would have decreased without the bundle over time, due to the improvement of intensive care and implementation of fast track procedures, for example. In addition, there were significant differences between the two groups in the administration of midazolam, opioids, and clonidine. The lower use of analgesia and sedation was not explicitly listed as a component in the delirium bundles (Figure 2). However, the association between high and prolonged doses of sedatives and analgesics and the occurrence of delirium was highlighted during staff training, so the reduced use of the medication may be attributed to this. A correlation of high-dose and prolonged use of sedatives and analgesics can be observed in our collective: Patients with positive delirium scoring had significantly higher use of opioids, benzodiazepines, and clonidine than patients without delirium. Pediatric delirium is related to the use of sedation medication, including benzodiazepines, opioids, propofol, and ketamine (31). Benzodiazepines have been shown to trigger or prolong delirium, especially in children (54). However, causality cannot be inferred from our data. Patients with critical illness, long ventilation time and long PICU stay are inevitably exposed to increased sedatives and analgesics due to the complex intensive care treatment. From our study, it is not possible to conclude the degree to which critical illness, complex intensive care treatment, and the use of medications contribute to the development of delirium. This raises the need for further research to better understand the risk factors for the development of delirium. The single-center design limited generalization to other PICUs. Another problem with pediatric delirium studies is the variation in delirium screening, which makes comparability difficult. The SOS-PD scale used in this study for assessment of pediatric delirium was validated in children between 3 months and 18 years of age admitted to a PICU. Patients with neurological abnormalities (e.g., encephalitis, coma) and periods of deep sedation (COMFORT behavior score <11) or neuromuscular blockade were excluded (25). To our knowledge, there was no delirium score available for children with developmental delay at the time of the study. Since the Cornell Assessment for Pediatric Delirium tool (CAPD) also has a limited informative value with children with developmental delay, Kaur et al. (55) found the combination of the CAPD with fluctuation in level of awareness over the course of a 24-h period as measured by the Richmond Agitation-Sedation Scale (RASS) to be valid and reliable for the diagnosis of delirium in children with developmental delay. This recent knowledge should be considered for future research, as a presumably relevant proportion of patients in a pediatric intensive care unit have diagnosed and undiagnosed developmental delay. We have not analyzed the data by delirium subtype. We must assume that some cases with hypoactive delirium were not detected. We cannot determine which measure of the PICU delirium bundle had the greatest impact on the decrease in delirium prevalence. Because the data were collected automatically using our patient data management system at the end of the observation period, we cannot exclude possible documentation errors. In addition, we observed a significant decrease in the frequency of scoring in the post-implementation phase. It confirms the conclusion of other authors that if protocols are implemented without training and regular monitoring of staff, there is a risk that quality will not be sustainably improved (56). Furthermore, it must be pointed out that compliance with the bundles was only checked on a random basis. Compliance could certainly be improved by continuous monitoring with the possibility of immediate intervention and motivation of the PICU staff. As also described by Ubeda Tikkanen et al. (57), we had significant problems during this study in diagnosing delirium in children with acquired brain injury due to overlapping symptoms of delirium and acquired brain injury.

Further studies are needed to understand the long-term effects of pediatric delirium and the impact on Post Intensive Care Syndrome (PICS), assessing medications, and their effect on development of delirium and to determine the efficacy and safety of interventions for delirium prevention and management in large randomized studies.

## Conclusion

Based on a validated delirium monitoring, our study gives new information regarding the prevalence of pediatric delirium and the characteristics of intensive care patients at risk for this significant complication. According to our data, the prevalence of delirium was reduced in a subgroup of pediatric intensive care patients after implementing non-pharmacological prevention and therapy measures. Especially young patients and patients after surgery for congenital heart disease seem to benefit from the implementation of delirium bundles. However, the overall delirium prevalence did not decrease significantly, and we cannot specify the impact of the improvement of critical care and change in PICU culture on this. Further research is needed for a better understanding. Based on our findings, it is important to promote change in pediatric intensive care—toward a comprehensive approach to prevent delirium in critically ill children as best as possible.

## Data Availability Statement

The raw data supporting the conclusions of this article will be made available by the authors, without undue reservation.

## Ethics Statement

The studies involving human participants were reviewed and approved by Ethics Committee of the University Hospital Tübingen. Written informed consent from the participants' legal guardian/next of kin was not required to participate in this study in accordance with the national legislation and the institutional requirements.

## Author Contributions

JM, ES, and FN designed the study, contributed to data collection, data analysis, and data interpretation. JM wrote the first draft of the manuscript. All authors contributed to the article and approved the submitted version.

## Funding

This study was supported by the non-profit foundation Hilfe für kranke Kinder. We acknowledge support by Open Access Publishing Fund of University of Tübingen.

## Conflict of Interest

The authors declare that the research was conducted in the absence of any commercial or financial relationships that could be construed as a potential conflict of interest.

## Publisher's Note

All claims expressed in this article are solely those of the authors and do not necessarily represent those of their affiliated organizations, or those of the publisher, the editors and the reviewers. Any product that may be evaluated in this article, or claim that may be made by its manufacturer, is not guaranteed or endorsed by the publisher.
