# Supplementary material for: Implementation of a Delirium Bundle for Pediatric Intensive Care Patients
Source: Front Pediatr. 2022 Feb 7;10:826259. doi: 10.3389/fped.2022.826259 (PMC8859429; doi:10.3389/fped.2022.826259)
Supplement: Supplementary file 1 [file Data_Sheet_1.PDF]

Universitätsklinik für
Kinder- und Jugendmedizin

Information about delirium and withdrawal from medication

Children's Intensive Care Unit 34

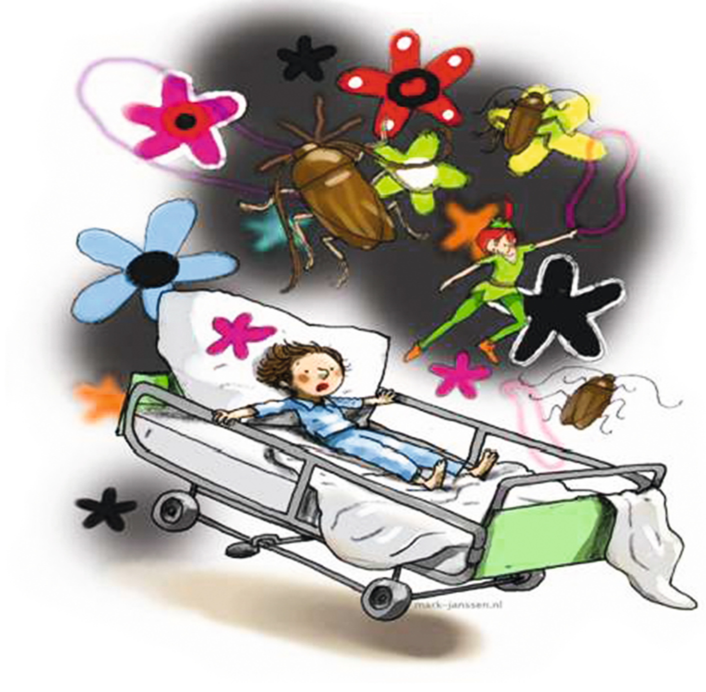

Universitätsklinikum
Tübingen

1 http://www.dimdi.de//static/de/klassi/icd-10-gm/kodesuche/onlinefassungen/htmlgm2014/block-f10-f19.htm Zugriff: 09.06.16

2 Neunhoeffer, F. (2015). „Entzug erkennen und behandeln“. Entzugssymptomatik und Delirium in der pädiatrischen Intensivmedizin . Tübingen, PPP

3 Schieveld, J. N., u.a. (2015). Pediatric Delirium, A practical approach. In J. Rey, IACAPAP Textbook of Child and Adolescent Mental Health (S. Kapitel I.5, 1-17). Geneva

4 Oldham MA, Lee HB, Desan PH.. Circadian rhythm disruption in the critically ill: an opportunity for improving outcomes. Crit Care Med 2016;44:207-17

Dear Parents and Relatives,

In this flyer, we would like to provide you with some important information about delirium and withdrawal from medication. This can occur during the period in the paediatric intensive care unit and the follow-up treatment.

Your team of the Children's Intensive Care Unit 34

What is withdrawal from medication?

Withdrawal from medication is a form of withdrawal syndrome in which several symptoms occur due to the discontinuation or reduced administration of sleeping medication and/or painkillers. The onset and course of the withdrawal syndrome is temporary. The manifestation depends on the drug in question and the dose which was previously administered.[1] The symptoms include shivering, sweating, nasal congestion, sneezing, yawning and vomiting, as well as an elevated temperature, an increased heart rate and increased blood pressure.

The withdrawal syndrome can occur in isolation but can also trigger or exacerbate delirium.[2]

What is delirium?

Delirium is a state of temporary confusion which can occur suddenly in severely ill children. Its symptoms include restlessness, a lack of eye contact, a low attention span and/or the child "living in its own world" and having unintelligible speech. A distinction is to be made between hyperactive delirium (in which the child is very active, restless, has hallucinations), and hypoactive delirium (in which the child is rather quiet, appears tired, and does not respond in the normal way when spoken to).

What can cause a case of delirium?

Delirium can have many causes. A child may become delirious due to a serious infection, a major operation (e.g., with a heart-lung machine), or a disease of the heart, lungs, or brain, for example. Changes to the usual sleep/awake rhythm can also lead to delirium. Withdrawal symptoms caused by the reduction or discontinuation of painkillers or sleeping medication can be another trigger.

In such cases, an individual tapering plan is drawn up to reduce the consumption of the medication by the child who has received it over an extended period of time. During the period in which the medication is tapered, a withdrawal and delirium assessment is completed. This is carried out by trained nurses on the basis of scales which are otherwise known as “scores”.

How long does a case of delirium last?

Delirium is temporary. As the child’s physical illness subsides, its confusion and unusual behaviour will also disappear.

What are the manifestations of delirium?

Children with acute confusion or delirium may exhibit the following behaviour:

- They do not think as clearly as they used to. Everything seems to pass them by, they do not realise where they are, and are not oriented to the time and place. In this situation, talking to them can be difficult, but it is still important.
- They often forget things that they have just been told. At the same time, they aren’t aware of their forgetfulness.
- As they may fail sometimes to recognize their surroundings, their reactions may be different from usual. The child may seem alert, suspicious, angry or even aggressive. They may often try to remove intravenous lines, feeding tubes,

monitoring instruments or bandages. Some children also withdraw into themselves, which may well be a form of behaviour that you have not witnessed before in your child.

- When suffering from delirium, children who are very sick may act as if they were much younger again. They may even temporarily appear to regress in the terms of their actual state of development. In such instances, it helps the child if you "meet them where they are", and behave towards them as if they really were younger.

- Delirious children may have a distorted perception of reality and see or hear things that do not exist, for example. At these moments, such things can seem very real to the child and can therefore be frightening. It is therefore better not to argue with your child about their perceptions, but also not to agree with them. The best approach is to support them with their reorientation and to explain repeatedly what is really happening and where they are.

- The child's symptoms can change frequently during the course of the day and often become stronger towards the evening. Periods of confusion may alternate with periods of calm and drowsiness.

Caring for children suffering from delirium

Caring for and treating children with delirium takes a lot of time and patience. Medication is available which can be used for the purpose of supportive, but no medication is available which can actually stop the delirium.

Children who are temporarily delirious have difficulty expressing themselves, communicating and responding emotionally. Being able to hear familiar voices and see familiar faces is especially helpful to them. This familiarity helps give them a feeling of security.

In particular, calm and confident behaviour on the part of the parents will have a calming effect on the child. In particular, with smaller children who are not yet able to speak, a loving, calm

approach is very helpful. This returns a sense of security and confidence to the little patient, even though the child may cry and refuse to become calm in the normal way.

You as parents and we as the treatment team can support your child with the following measures:

- Speak calmly, and in clear, short sentences, even if your child is not yet able to talk.
- Tell your child who you are and why you are there. Repeat this if necessary.
- If you have an older child, you can help with their orientation by telling them where you are right now (hospital, ward) and what day or time of day it is, so that they can orientate themselves to the time and place.
- If your child is awake, sit them up in bed, if possible, so that they can look at their surroundings. Tell them who the people in the room are and what they are doing.
- Bring along photos of your home, family and friends if available. Put the photos on display so that your child can see them.
- It helps your child if you ask them simple questions to which there is only one answer: “Yes” or “No” – for example, “Did you sleep well?” instead of “Did you sleep well, or did you wake up a lot?”
- If too many questions are asked, your child will become confused. Most of the time, it is sufficient and valuable for your child if you, as an important caregiver, are simply there.
- Visits are very important for your child, but it is important that there should not be too many visitors at the bedside at once. Only two visitors should be with your child at the same time.

It is then best to stand on the same side of the bed so that your child is able to concentrate better.

- Make sure that your child wears their glasses, hearing aids, etc. so they can perceive their surroundings as effectively as possible.

- If your child complains of seeing or hearing unusual or frightening things, it is better to point out to them that these are only dreams and not reality. However, do not argue with your child, just make it clear to them that you understand the situation differently. With older children, try to direct the topic towards current events or the people who are in the room.[3] With smaller children, as a parent, you can also “chase away bad things”, because a smaller child will trust its parents to be able do this.

Sleep is very important in preventing or reducing delirium. We will try to give your child as much rest as possible at night by reducing the light and noise – your child may be given earplugs. It usually takes a few days for your child to get back into his or her usual day/night rhythm if it has previously been given sleeping pills and painkillers for several days or has been on a ventilator. The administration of the sleep hormone melatonin can also provide support here. This will help your child to fall asleep and stay asleep.[4]

If a child is confused, precautions must be taken to protect them from self-harming behaviour. In the case of considerable restlessness, it may be necessary to restrict your child’s mobility for its own protection, which is known as restraining. Velcro cuffs are attached to your child’s hands so that it is still able to move but cannot reach important access points such as the breathing tube in the trachea (tube) or the central venous line (CVC). Bed restraints can also be used on older children and adolescents if there is a risk that they could fall out of bed.

Use of occupational therapists

As described above, parental involvement plays a very important role in the treatment of delirium. However, your continuous presence in the paediatric intensive care unit is not possible for you as a parent. To be able to support your child according to their needs, we use occupational therapists in addition to our nursing staff.

What are the tasks of the occupational therapist?

With their specific training and education, the occupational therapist supports both your child and you as parents during the intensive care in the role of trusted caregivers. If there is a period of delirium, they will help to reduce and ideally prevent the symptoms. To this end, the occupational therapists:

- guide and accompany you as parents and relatives in managing delirium in children
- ensure the child receives attention from known persons
- encourage the orientation of your child
- encourage your child's independence and the expression of its needs
- support the organisation of auxiliary products such as glasses and hearing aids
- take shielding measures and ensure a calm environment for children suffering from hyperactive delirium through the reduction of auditory and visual stimuli, e.g. the reduction of alarm volume levels and mobile phone sounds
- encourage exercise and coordination
- carry out memory training and reading out loud

Our specially trained occupational therapists support you and your child via a project financed by donations of the foundation HILFE FÜR KRANKE KINDER and are looking forward to answering any questions you might have.

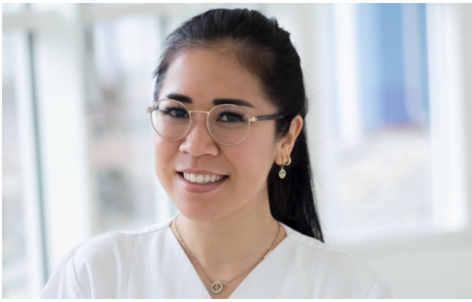

Susy Hinderberger

Occupational therapist

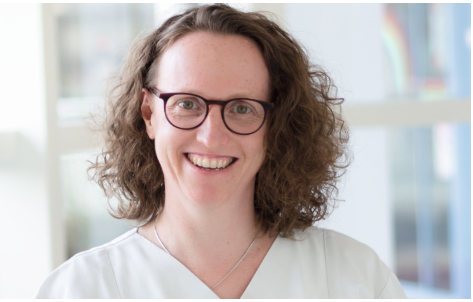

Sr. Emanuela Koch

Occupational therapist

We hope that this information has given you a helpful insight into the clinical picture of delirium, as the support you give your child as parents is of considerable importance to their recovery.

If you have any further questions, please contact the nurse, occupational therapist or doctor responsible for your child.

Thank you

The printing of this brochure was made possible by donations to HILFE FÜR KRANKE KINDER - Die Stiftung in der Uni-Kinderklinik Tübingen.

Donation account: DE61 6415 0020 0000 5548 55 (BIC: SOLADES1TUB)

www.hilfe-fuer-kranke-kinder.de

Hilfe für kranke Kinder
Die Stiftung in der Uni-Kinderklinik Tübingen

Impressum

Herausgeber
Universitätsklinik für Kinder- und Jugendmedizin Tübingen

Verantwortlich für den Inhalt
Prof. Dr. Michael Hofbeck, Ärztl. Direktor Kinderkardiologie, Pulmologie, Intensivmedizin

Text
Gertrud Kappe, Clivia Langer, Dr. Felix Neunhoeffer

Bildnachweis
Abbildung Titelseite: Child with Pediatric Delirium. Mark Janssen, mit Erlaubnis
Fotos: Karoline Niethammer, Philipp Nährig

© 2021 Universitätsklinikum Tübingen

Kontakt

Universitätsklinik für Kinder- und Jugendmedizin
Station 34 / Kinderintensivstation
Hoppe-Seyler-Str. 1
72076 Tübingen
Telefon: 07071 29-87173

www.medizin.uni-tuebingen.de/kinderklinik
